# Clinical significance of genomic sequencing of circulating tumour cells (CTCs) in cancer

**DOI:** 10.1016/j.jlb.2023.100135

**Published:** 2023-12-28

**Authors:** Abdul Auwal, M. Matakabbir Hossain, Tasfik Ul Haque Pronoy, K.M. Rashel, Md Nurujjaman, Alfred KY. Lam, Farhadul Islam

**Affiliations:** a Department of Biochemistry and Molecular Biology, University of Rajshahi, Rajshahi, 6250, Bangladesh; b School of Medicine and Dentistry, Gold Coast Campus, Griffith University, Gold Coast, QLD, 4222, Australia

**Keywords:** Circulating tumour cells, Single cell, Survival rate, Diagnosis, Prognosis, Genome sequencing

## Abstract

Circulating tumour cell (CTC), a rare subpopulations of tumour cells, plays a significant role in cancer metastasis and recurrence. The current review focuses on information of CTCs detection, enrichment, genome sequencing and investigating on clinical significance of CTCs sequencing in monitoring progress of patients with cancer. Tissue biopsy is not always favorable for monitoring cancer recurrence and metastases and can be difficult and risky for the patient’s condition. On the other hand, enrichment and characterization of CTC could be effective in these circumstances. Accordingly, a number of detection (physical, immunological etc.), isolation (laser capture microdissection, DEPArray Di Electro phenetic array, fluorescence-activated cell sorting etc.) and enrichment platforms (Cellsearch, MagSwepeer, CTC-Chip etc.) have been developed. In addition, technologies for phenotypic characterization and genomic profiling (Tang’s method, Smart-seq, Cel-seq etc.) at single-cell level has being established followed by genomic amplification. Importantly, numerous preclinical and clinical studies showed effective prognostic and predictive implications of molecular characterization of CTCs. CTC’s sequencing has been successfully used as an effective tool to monitor genomic variations in the primary and metastatic tumours, thereby could predict the therapy resistance, recurrence of tumours. The genes expression profiles, stratification of cancers as well as identify the cancer cells with potential to undergo epithelial-mesenchymal transition (EMT) could also be identified. In addition, detection of mutational variants of CTCs by genome sequencing infer the therapeutic outcome and patient’s survival. Therefore, CTC sequencing has potential to be used as a liquid biopsy tool for management of patients with cancers in clinical settings.

## Highlights

• Development of enrichment and isolation of CTC for various cancers
• Technologies for phenotypic characterization and genomic profiling at single-cell level
• Effective prognostic and predictive implications of molecular characterization of CTCs
• CTC’s sequencing could predict the therapy resistance, recurrence of tumours
• Detection of mutational variants of CTCs by genome sequencing infer the therapeutic outcome and patient’s survival

## Graphical abstract

**Figure:**
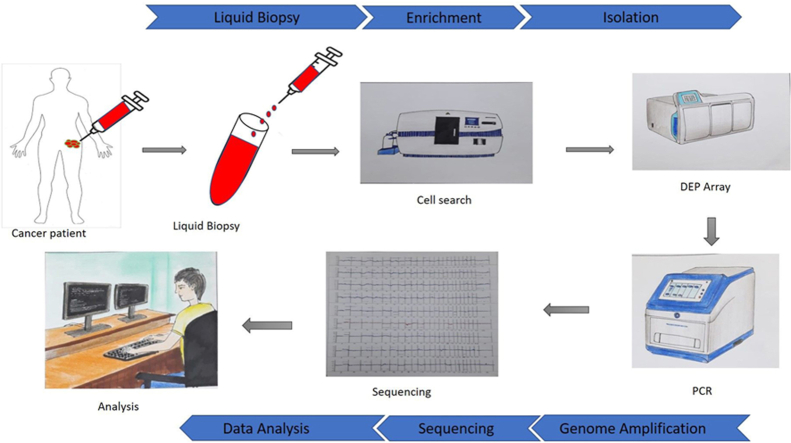

## 1 Introduction

Cancer metastasis is the key contributor of cancer related deaths. The cancer cells involved in tumour spreading reach the metastatic sites from the primary site through the circulating system [1]. Circulating tumour cells (CTCs) are tumour cells that shed from the primary tumour and intravasate into the peripheral blood circulating system. They have overwhelmed antigenic and genetic characteristics of the specific type of original tumour, which could be responsible for the metastasis [2]. During the metastatic process, they detach from the tumour tissues, which then circulate in the bloodstream and extravasate when they reach distant sites (the estimated time is about hours to days) and developed secondary tumours followed by colonization. These CTCs could reside in the blood as a single cell or cluster of cells [3]. The development of functioning CTCs are extremely uncommon event. The detection, extraction, enumeration, and characterization of CTCs are technically difficult as they are in small numbers when compared to the billions of erythrocytes and millions of leucocytes in a milliliter of blood [1]. In 1869, Australian pathologist Thomas Ashworth first observed CTC in the blood of a patient with metastatic cancer [4]. It was not until more than 100 years later, that the critical roles of CTCs in the metastatic spread of carcinomas were demonstrated followed by the development of sensitive and reproductive advanced technologies, which explore the clinical significance of CTCs in patients with cancers [3].

The isolation and identification of CTCs from other biological fluids such as pleural fluid (PF), cerebrospinal fluid, or urine have the potential to be used as the starting material, thus, have important implications for cancer diagnosis and prognosis [5]. These biological fluids hold great significance as they can lead to the discovery of new CTC origins and further the understanding of CTC clusters in metastatic malignancies [5]. In comparison to peripheral blood, malignant pleural and peritoneal effusions are a richer source of CTCs and CTC clusters. A buildup of PF in the pleural space may result from the advancement of certain cancers, most commonly NSCLC, breast cancer, Kaposi sarcoma, and lymphoma [[6], [7], [8]]. For example, CTCs from PF in patients with NSCLC could showed mirrored features those from blood samples [9]. Similarly, CTCs in the CSF of patients with leptomeningeal metastasis (LM) could be detected using the rare cell capture technique (RCCT) with great sensitivity and specificity, which might be attributable to the RCCT method of immunomagnetic enrichment and its capacity to exclude EpCAM ​+ ​leukocytes [10]. As a result, it has been demonstrated that counting CTCs in CSF allows for a more precise estimation of tumor burden than does traditional CSF cytology [11], thereby avoiding the drawbacks of techniques reliant on tissue biopsy and neuroimaging [12]. In addition, bladder cancer might be diagnosed and managed with beter care if the need for a painful endoscopy is avoided and the tumor cells, especially CTCs found in urine can be captured with sensitivity [13].

The relationship between the number of CTCs and disease progression has been revealed with the counting of CTCs in patients with metastatic carcinomas and poor patient prognosis is associated with increased count of CTCs in peripheral blood in many cancers [[14], [15], [16], [17], [18]]

Furthermore, CTCs can be detected in the blood before, and after treatment and during treatment to track patients' responses to therapy, allowing physicians to monitor the disease condition [14]. Tissue biopsy is not always favorable for monitoring cancer recurrence and metastases and can be difficult and risky for the patient’s condition. Thus, CTCs could offer a fresh perspective for comprehending the molecular process of tumour occurrence, and progression and could be a potential surrogate biomarker.

The understanding of cancer metastasis and the events associated with tumour progression could be facilitated by the transition of a primary tumour to a metastatic tumour, which is driven through the mutational evolutionary process [19]. Thus, genomic analysis of CTCs, especially single CTCs, could provide insights to uncover clinically relevant information. However, due to the low number of CTCs in the bloodstream (as few as 1 CTC per 10^6^ or 10^7^ leukocytes), the detection and characterization of CTCs are technically challenging. Also, CTC extracted is unlikely to be suitable for effective high-throughput investigations such as whole genome sequencing (WGS) [20]. On the other hand, there is robust development of bioinformatics and sequencing technologies, deep sequencing, for instance, can find alterations in genes and noncoding DNA (including regulatory sequences). These methods could provide information regarding the alteration connected with disease phenotypes and the use of prospective therapeutic targets (Childs et al., 2015).

Single-cell sequencing of CTCs can be used to examine the heterogeneity of CTCs, differences between single-cell genomes, transcriptomes, and epigenetics in peripheral blood CTCs, primary tumour and metastatic foci, and metastatic lymph node cells. Accordingly, single-cell sequencing has been employed in the study of numerous cancers such as breast, bowel, lung, prostate cancers, melanomas, and other cancers [[21], [22], [23], [24]]

**Fig. 1:**
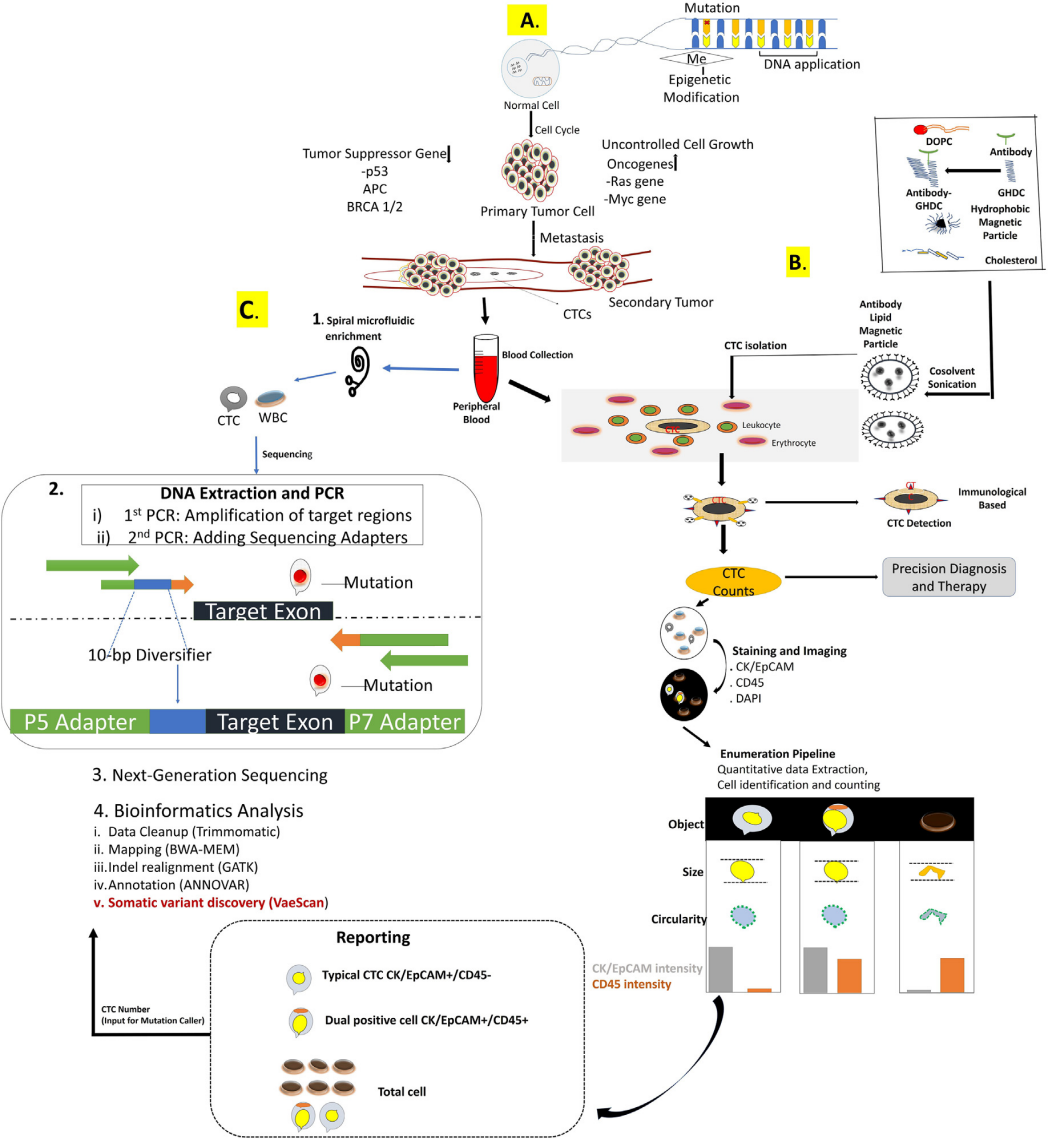
**Genesis, physical characterization, and single-cell CTC analysis in clinical applications**. **A**. Biogenesis of CTCs. Abbreviation of the nucleotide sequence can be activated oncogenes and inactivation of tumour suppressor genes lead to uncontrolled growth of cancer cells. Through the process of metastasis, free tumour cells detached from the primary tumour that travel in the bloodstream that we know as circulating tumour cells. **B**. Antibody Preparation, CTC isolation, Detection, Phenotypical Characteristics. The reaction between antibodies and GHDC formed Antibody-GHDC compound derivative, and then magnetic immunoliposomes (MILs) are made by a reversed-phase one-step method combining DOPC, Cholesterol, and Fe3O4-HMN. Making MILs is a useful method for separating tumour cells. After blood collection from the patient, the prepared antibody (lipid magnetic particle) isolated the CTCs and can be detected with the EpCAM-based immunological method. The phenotypical characteristics are observed through the immunostaining process. **C**. CTC enrichment, DNA Amplification, Next Generation Sequencing (NGS). After blood collection, CTC is enriched through a spiral microfluidic chip system, which utilizes inherent centrifugal force in the spiral microchannel to deplete white blood cells (WBCs) and enrich CTCs based on their cell sizes. NGS library preparation is simple and only requires two PCR procedures to create the library. The generated amplicon libraries include three components: a 10-bp diversifier sequence, P5 and P7 adapters for binding to the flow cell, and a targeted exon region.

Although CTCs have shown considerable promise in clinical cancer diagnoses, their use as a surrogate biomarker for cancer screening, treatment monitoring, and prognosis prediction remains limited. Depth, characterization of CTCs could provide better clinical insights into the patient’s genetic makeup, which in turn can guide the clinicians for better management of patients with cancer in clinical settings (Fig. 1).

## 2 Biology of CTC

**Fig. 2:**
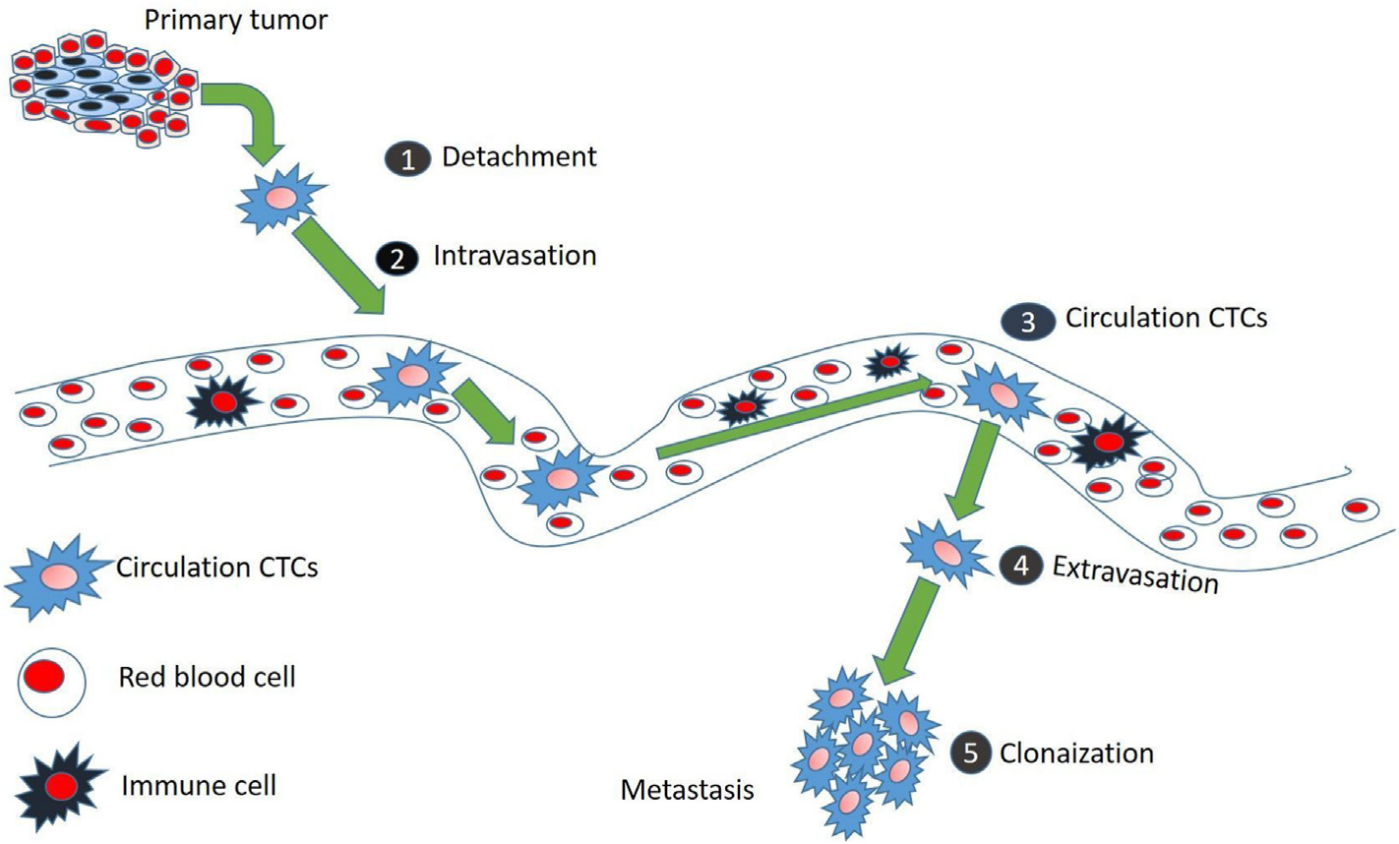
**Roles of CTC in cancer metastasis.** Cancer metastasis is a multistep process. Detachment from the originating site, invasion, and intravasation, into the bloodstream, survival as CTCs and interaction with the blood cells, extravasation from the bloodstream and attachment to and colonization of the metastatic site are all components of the intricate metastasis process.

CTCs are a subset of tumour cells that separate from a solid tumour, are highly active, and have a high potential for metastasizing at the secondary sites traveling through the peripheral blood (Fig. 2). The status of a primary tumour is correlated with the quantity and characteristics of CTCs. The biological characteristics of metastatic tumour can be gained by counting and examining the properties of CTCs [21]. CTCs derived from various cancers including large bowel, breast, lung, hepatic, prostate, pancreatic, etc., had EpCAM, a "universal" epithelial cancer marker on their surface [25]. In circulation, CTCs can exist in two forms such as single CTCs or in clusters of CTCs, which is also known as circulating tumour microemboli (CTM). CTMs can play an important role in cancer metastasis as they can exhibit resistance to cell death in circulation, thereby contributing more to micro-metastasis compared to single CTCs [26].

Accumulating information indicated that there is substantial genetic and behavioral variability in CTCs [2]. According to the morphologic characteristics, single CTCs are generally classified into three subpopulations such as epithelial circulating tumour cells (ECTCs), epithelial–mesenchymal circulating tumour cells (EMCTCs), and mesenchymal circulating tumour cells (MCTCs) [27]. The physical features of CTCs are yet to be established. However, it is suggested that they are larger in size when compared to leukocytes. Also, they are nucleated and express specific surface markers. Furthermore, they are highly plastic with an enlarged nucleus (more than 8 μm–10 μm), implying that they have high transcriptional activity, thereby having the potential to survive under different physiological conditions in the circulation [26]. Moreover, CTCs can express biomarkers, including human epidermal growth factor receptor-2 (HER2), estrogen receptor, prostate-specific membrane antigen, folate receptor, and survival in various malignancies (Table 1). Genetically, many CTCs are aneuploidy with various chromosomal abnormalities, depending on the neoplastic origin of CTCs [2]. For instance, CTCs from breast cancer patients showed phosphorylated receptors, including epidermal growth factor receptor (EGFR), human epidermal growth factor receptor (HER)2, phosphoinositide-3 kinase (PI3K), phosphorylated-focal adhesion kinase (pFAK), Anna Kaiser Technique (AKt), and vascular endothelial growth factor (VEGF), which may contribute to their proliferative and survival benefits [2,28,29]

**Table 1:** Molecular markers used to identify CTCs in various type of cancers.

Types of cancer	Biological marker of CTC	Sensitivity	Speacificity	References
Breast carcinoma	EpCAM/CK8,18,19; Vimentin; HER2; CK 5/7/8/18/19; Twist; ER; E-Cadherin; Fibronectin; AR; MRP	76.1 %	92.3 %	[75]
Prostate carcinoma	EpCAM/CK8,18,19; Vimentin; PSMA; Twist; PSA; EGFR; ARV7; PIM1; AR v567es, PanCK, CD45, PSMA, AMACR,	EpCAM,95 % PanCK,95 % CD4595 % PSMA,92.5 % AMACR,92.5 %	100 % 100 % 100 % 100 % 100 %	[87]
Renal cell carcinoma	EpCAM; CD147; EMT; CA9/CAIX.	35 %	100 %	[88]
Bladder carcinoma	EpCAM/CK8,18,19	35.1 %	89.4 %	[89]
Colorectal carcinoma	EpCAM/CK8,18,19; Vimentin; PI3Kα; Twist; CEA; CYFRA 21; CYFRA 21-1; SNAI1; PRL3; AKT2; LOXL3; Plastin3.	69.6 %	95 %	[90]
Small-cell lung carcinoma	EpCAM/CK8,18,19; Vimentin; DLL3	72 %	96 %	[91]
Non-small-cell lung carcinoma	CK7/8/18/19; Vimentin; Folate receptor; EpCAM/CK8,18,19; Twist; Telomerase activity; N-Cadherin; AXL. NSE;	51 %	87.5 %	[92]
Hepatocellular carcinoma	EpCAM/CK8,18,19; Vimentin; GPC3; Twist; ASGPR; GNB4	ASGPR-97.8 % EpCAM-97.8 % GNB4-88.2 %	100 % 100 % 100 %	[93]
Pancreatic carcinoma	EpCAM/CK8,18,19; Vimentin; CA19-9; ICAM-1; OPG; CEA;	CA19-9, ICAM-1,OPG-78 % cathepsin D, MMP-7-88 % CEA-98 %	94 % 80 % 93.8 %	[94]
Gastric carcinoma	EpCAM/CK; Vimentin; XAF1; CK19; CK20; CEA; N-Cadherin; MT1-MMP; Survivin; HER2.	CK19 27 % CK20-25 % CEA-31 %	95 % 95 % 94 %	[95]

Although the CTCs are extremely heterogeneous in regards to molecular make-up, a degree of similarity is also conceivable, indicating the genetic alterations contributed to cancer cells to execute CTC-like activities such as invasion, traveling into the circulation, and extravasation, resulting in cancer metastasis [30]. For example, CTCs isolated from patients with breast cancer showed signatures of recurrent gain of 90 minimal common regions (MCRs), predominantly located on chromosome 19 [30]. Among these CTC genomic signatures, one cluster (16 MCRs; AKT2, PTEN, CADM2) is associated with dormancy whereas another cluster (358 MCRs; ANGPTL4, BSG, MIR-373) correlated with the aggressiveness of CTCs. In addition, two common MCRs (19q13.13 and 21q21) were noted, which are responsible for resistance to anoikis, TGFβ-signalling, and metastasis (TFF3, LTBP4, NUMB) in CTCs [30]. Another study noted that CTCs from patients with metastatic breast cancer had amplification in the cyclin D1 locus [22] Whole-exome sequencing of CTCs isolated from patients with metastatic prostate cancer showed 10 early trunk and 56 metastatic trunk alterations [31]. Therefore, CTCs from different cancers can harbor different genetic signatures, which could be used as targets for developing new therapeutics for the selective elimination of CTCs, thereby preventing cancer cell dissemination and metastasis.

## 3 CTC detection

**Fig. 3:**
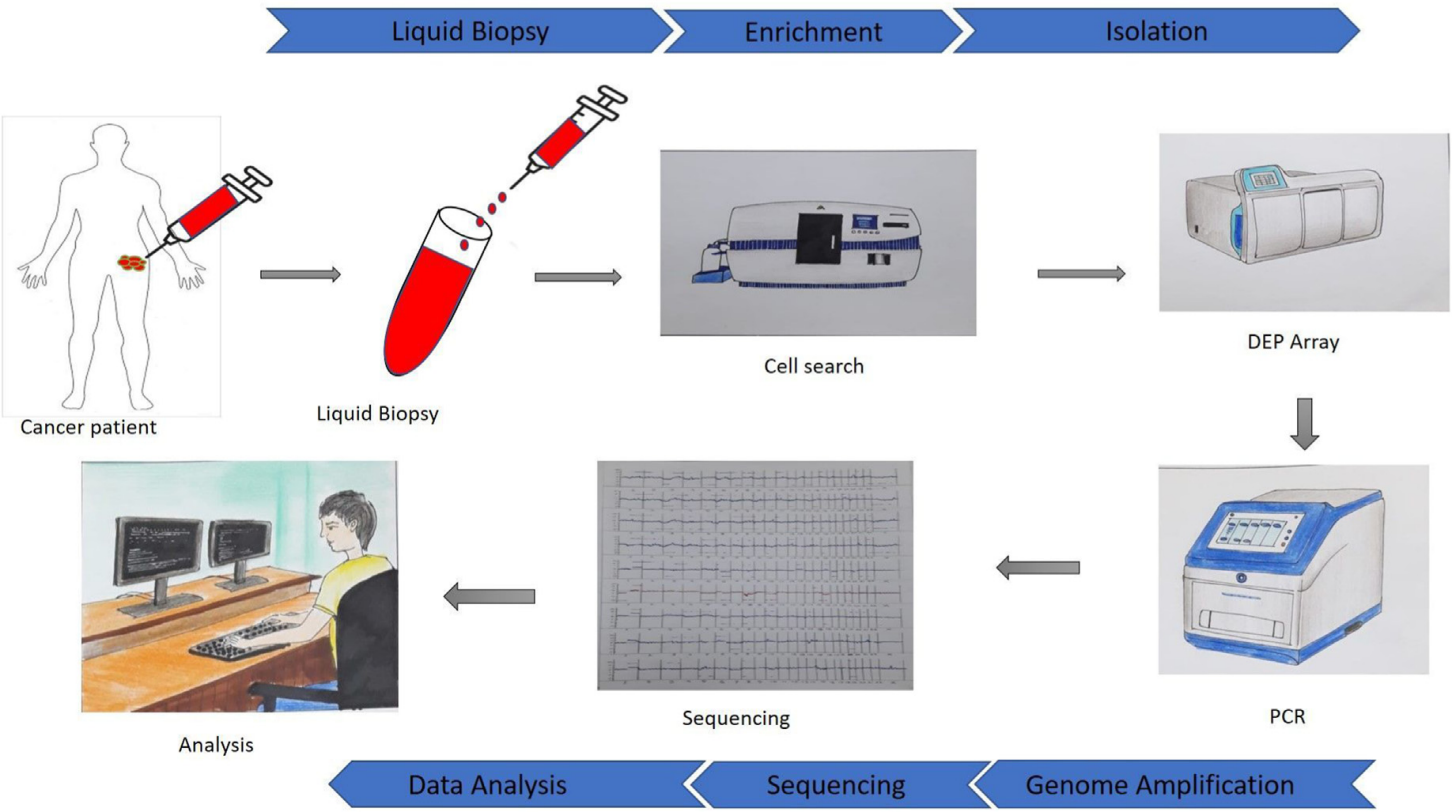
**CTC sequencing analysis workflow.** Single-cell CTC sequencing analysis is carried out in six steps such as sample collection (lipid biopsy), enrichment, isolation, genome amplification, sequencing, and analysis. First, peripheral blood is collected from patients with cancer with a syringe and stored in coated vials. Rare CTCs in peripheral blood are captured by enrichment methods such as cell search. DEPArray allows the isolation and detection of CTCs from peripheral blood at the single cell level. This CTC's genome (DNA and RNA) is amplified by different PCR amplification methods. Finally, numerous methods (hydroseq, EPISPOT, and EPIDROP) are used for CTC sequencing and analysis by computerized methods.

Difficulties in obtaining a biopsy on many internal organs limit the use of biopsy specimens for the detection of metastatic malignancies. This shortcoming could be overcome in clinical settings using liquid biopsies to detect CTCs present in the peripheral blood of patients with cancers. CTC could be found in peripheral blood even if the cancer is in the early stage. However, it is challenging to detect CTCs in patients with early stages of primary cancer or advanced stages of metastatic cancer. Thus, appropriate techniques are required for the detection and enrichment of rare and low concentrations of CTC detection as only a small number of CTC could be present in the peripheral blood collected for analysis [32]. There are several CTC detection platforms and strategies have been established for the heterogeneous CTCs isolation, separation, and characterization (Fig. 3). The detection of CTCs can be achieved through enrichment or without enrichment.

### 3.1 CTC detection with enrichment technologies

The CTCs enrichment techniques can broadly be divided into positive enrichment methods that are based on capturing CTCs amongst healthy blood cells and negative enrichment methods that elute CTCs by removing non-target cells (such as RBC and WBC) [33,34]. Principally, these methods use antibodies to differentiate cells based on epithelial cell surface markers expressed on CTCs (Table 2).

**Table 2:** CTC enrichment technology with their advantage and disadvantage.

Enrichment Technology	Category names	Advantages	Disadvantages
Cellsearch	Immunomagnetic positive enrichment	Semiautomated can process up 8 sample at a time	Recovery of EpCAM^+^CTCs only. High levels of EpCAM expressing CTCs cell can be detected
MACS		High recovery; High purity rates	Recovery of EpCAM^+^CTCs only
MagSweeper			
IMS			
Easysep	Immunomagnetic negative enrichment	Recovery of heterogeneous population of CTCs	Loss of CTCs aggregates surrounded by white blood cells
RosetteSep			
CTC-Chip	Microfluidic immunocapture positive enrichment	High purity rate; High capture efficiency; High cell viability	Long, time-consuming process.
HB-Chip			
Nanovelcro			
Isoflux			
FMSA	Size base enrichment	Quick and non-specific	Inability to distinguish monocytes from CTC.
Screencell			
ISET			
OncoQuick	Density base	inexpensive	Loss of very small CTCs and cell aggregates
Accucyte			
MCA	Microfluidics	Recovery of heterogeneous population of CTCs. Ability to capture CTC cluster	Slow processing time on-chip imaging difficult

EPCAM: epithelial cell adhesion molecule ISET: isolation by the size of epithelial tumour, MACS: magnetic activated cell sorting, IMS: immunomagnetic separation, FMSA: the flexible micro spring array, MCA: microfluidic colorimeter analysis.

#### 3.1.1 Immunomagnetic-based positive enrichment

CTCs detection by positive enrichment using immunomagnetic assays carried out both in vivo and in vitro. For example, in vivo CellCollector® (Gilupi, Germany) method is developed for the isolation of CTCs [35]. The CellCollector consists of a gold-coated steel wire with a polymer on top holding anti-EpCAM antibodies. The antibodies-coated wire is placed into the cubital arm vein for 30 min and the attached cells are then stained, counted, and morphologically characterized [35] This method can analyze large blood volumes and could enable earlier detection of rare CTCs, even during the therapies [35]. However, there are practical limitations that arise in a low amount of blood (5–10 ml) analyzed ex vivo that could be drawn from a patient [35] In addition, Vermesh and colleagues established an in vivo MagWIRE (magnetic wire) system for intravascular CTCs retrieval and enrichment using functionalized magnetic beads [36]. They used a flexible magnetic wire with antibody-coated magnetic particles that capture CTCs from entire blood with 500–5000 times greater capture efficiencies than the commercially available CellCollector (Gilupi, Dűsseldorf, Germany) and 10–80 times greater than the in vitro methods uses 5 ml blood drawn from peripheral blood [36]. However, the magnetic beads must be intravenously given throughout the assay. Thus, further investigation is still needed to confirm its efficacy in clinical applications.

A few in vitro enrichment methods can capture the rare CTCs in peripheral blood samples drawn from patients with cancers [[37], [38], [39], [40]]. For example, CellSearch (CellSearch, Huntington Valley, Pennsylvania, USA), the only Food and Drug Administration (FDA) approved method for CTCs detection, uses a 7.5 mL peripheral blood using magnetic nanoparticles coated with antibodies that target epithelial cell adhesion molecules. After CTCs are magnetically extracted, multiplex labeling is performed using fluorescence-labeled monoclonal antibodies for leukocytes (CD45), epithelial cells (CK8/18/19), and a nuclear dye (e.g., DAPI). CTCs are then identified and characterized using FACs (Fluorescence-activated cell sorting) based on their expression of cytokeratins (CK), EpCAM, and lack of expression of CD45 [41]. Also, Magnetic Activated Cell Sorting (MACS) could be used for CTC detection in vitro using magnetic beads labeled with an antibody against cytokeratins (Pan-CK; e. g. CK7, CK8, CK18, CK19) and EpCAM) [38]. Another magnetic cell-sorting platform, namely MagSweeper can perform the enrichment using magnetic beads coated with the monoclonal BerEP4 antibody against human EpCAM [39]. The CTCs that have been magnetically captured are subsequently drawn with sheath-covered magnetic rods as they pass through the capture wells. This system successfully purified CTCs from patients with metastatic breast cancer with high efficiency [39]. Furthermore, Xiong et al. established an in vitro biomimetic immuno-magnetosomes (IMSs) system for the high-performance enrichment of CTCs [40]. They used Fe_3_O_4_ magnetic nanoclusters (MNCs) covered with leukocyte membrane fragments (LMNC) through electrostatic interaction to produce magnetosomes that repel leukocytes in peripheral blood due to their homology and unspecific leukocytes are absorbed. They reported 90 % detection and isolation efficiency of CTCs from peripheral blood in patients with cancers [40].

#### 3.1.2 Immunomagnetic-based negative enrichment

Immunomagnetic negative enrichment focuses on capturing leukocytes using magnetic beads coated with anti-leukocyte antibodies (anti-CD45) [41]. Negative enrichment techniques are advantageous for facilitating the separation of CTCs without tempering or manipulating the expression of any cell surface markers of CTCs and, thus, may have a broad range of clinical applications. EasySep™ (StemCell™ Technologies, Vancouver, British Columbia, Canada) is a widely used immunomagnetic-based negative enrichment method that uses antibodies to recognize leukocytes and unwanted cells isolated as negative depletion [9]. CTCs were detected and isolated in various cancers with a range of efficiencies (44 % in colon cancer, 50 % in ovarian cancer, 80 % in gastric cancer, and 100 % in lung cancer) using this method [42].

RosetteSep™ CTC enrichment is another negative selection method using a cocktail combined with the ficoll-gradient centrifugation containing the anti-CD36 antibody, which is designed to enrich circulating epithelial tumour cells from fresh whole blood [42]. In this system, tetrameric antibody complexes are used to recognize red blood cell markers such as CD2, CD16, CD19, CD36, CD38, CD45, CD66b, and glycophorin A. After centrifugation using a density gradient medium such as Lymphoprep™, Ficoll-Paque, or STEMCELL Technologies, antibody-labeled cells are settled to the bottom with red blood cells [21,43]. RosetteSep™ is also used in combination with EasySep™ methods to remove unwanted cells.

Another method, namely puncher technology, is an isolating technology of single cells, which leads to high recovery rates of single CTCs from a small number of enriched samples with 80–90 % efficiency [44].

### 3.2 CTC detection without enrichment technologies

Researchers have also developed several platforms for CTCs detection and isolation without enrichment, which are known as direct detection of CTCs.

Line-confocal microscopy is an automated high-throughput cell counting method based on microfluidics and line-confocal microscopy [45].

Multiple antibodies conjugated with different fluorophores are applied to label the peripheral blood. Then the labeled blood sample is pumped by hydraulic pressure through a microfluidic channel and analyzed with a line-confocal microscope. In this way, CTCs are automatically counted and reported based on fluorescence signals and labelling schemes. The clinical results from a study using a line-confocal microscope flow counting system found a median of 90 CTCs per 7.5 mL of blood compared to a median of zero for the CellSearch system [45].

Surface-enhanced Raman Spectroscopy (SERS) is a technique for directly detecting and counting CTCs in the presence of white blood cells by nanoparticles conjugated with epidermal growth factor (EGF) peptide as a targeting ligand [46,47]. Bio-conjugation of EGF-SERS nanoparticles encoded with Raman reporter molecules (QSY), which adsorbed negatively charged Au-nanoparticle surface via electrostatic interaction. It was reported that this platform can successfully identify CTCs with a range of 1–720 per milliliter of whole blood in patients with squamous cell carcinoma of the head and neck [47]. Furthermore, the Nanovelcro chip, which is composed of a nanofiber cell affinity substrate and overlaid chaotic mixer was used in conjunction with a laser capture microdissection microscope to isolate and capture the CTCs [47].

Di-Electro-Phonetic Array system (DEPArray), a novel semi-automated single cell-sorting platform, allows the isolation and detection of CTCs from peripheral blood at the single cell level [48]. The accuracy of cell selection at low cost is high, however, this method takes a long time with low flux, and it easily causes mechanical damage to the target cells, which limits the subsequent clinical applications [49,50].

Overall, researchers developed several platforms and strategies to detect, isolate, and enrichment of CTCs for various cancers. However, these methods have some limitations and require further improvement for clinical applications (Table 3).

**Table 3:** Advantages and disadvantages of various CTC isolation methods.

Methods	Advantage	Disadvantage
LCM	Observation of cellular morphology and physiology to prevent possible contamination or cell damage. LCM also allows an accurate target cell recovery.	Time Consuming and labour intensive.
FACS	FACS act as high throughput manner and it isolates specific individual cells with correct markers	Requirement for many input cells.
Microfluidic separation	High flux, small reaction volume, less space, and less pollution	High cost and high cell loss rate
Puncher Technology	High recovery rates and isolate a single CTC accurately from a small quantity.	Success rate approximately 80–90 %
DEPArray	separates rare cells from a mixed cell population, semi-automated	the long time required to isolate the cells
CellCelector system	Keeping cell activity, high accuracy, and less time consuming	High cost and high instrument dependence
Microoperation separation method	High accuracy and low cost	Time consuming, low flux easy to cause mechanical damage to the target cells

LCM: Laser capture microdissection, FACS: Flow Cytometry Analysis, DEPArray Di Electro phenetic array.

## 4 Sequencing of CTC

The molecular characterization of tumours can provide detailed insights for better detection of metastases, prediction of prognosis, and planning targeted therapies for patients with cancers. Therefore, clinical oncology researchers focus on the investigation of CTCs genome by sequencing for a better understanding of the molecular makeup, and heterogeneity of CTCs in inter- and intra-tumours [51,52]. The high-throughput whole genome amplification, next-generation, and deep sequencing technologies permit genomic analysis at the single cell level that is uniquely placed to unravel complex clonal make-up and track the clonal evolution of tumour cells over time [52]. In addition, CTCs genome sequencing helps in examining gene expression as well as the mechanisms of the epithelial mesenchymal transition properties of CTCs [53]. The following sections illustrate the genome or transcriptome amplification and sequencing of CTCs.

### 4.1 Genome amplification

The genomic content (DNA & RNA) of a cell is only 6–7 pictograms, thus, the genetic material needs to be amplified by high throughput sequencing platforms that required high-quality and much larger scale as starting materials [44,53,54]. During the last decade two amplifying platforms, Whole Genome Amplification (WGA) and Whole Transcriptome Amplification (WTA) have been developed [54]. Multiple displacement amplification (MDA), multiple annealing and looping-based amplification cycle (MALBAC), and polymerase chain reaction (PCR) are usually used as amplification methods in single-cell studies (SCS) [53]. These methods have both advantages and disadvantages as outlined in Table 4. Whereas Cell Expression by Linear amplification and sequencing (CEL-Seq), Single-cell tagged reverse transcription sequencing (STRT- Seq), and Switching Mechanism at the end of the 5′-end of the RNA Transcript (SMART- Seq) analysis are the common approaches for whole transcriptome amplification [55,56]. These approaches were developed to amplify either full-length transcripts or their 3′ region. The cDNA library generated by these techniques was used for WTA analysis of CTCs [57]. Table 5 shows the methods commonly used for CTC sequencing.

**Table 4:** Methods for whole Genome amplification for CTC analysis.

Methods	Advantage	Disadvantage
MDA	High coverage and uniformity, good accuracy long amplicons	High allele deletion rates, exponential amplification causes sequence dependent deviations, not suitable for detecting copy number variation (CNV)
MALBAC	High and uniform coverage of the genome 93 %, use for copy number variation	High false positive rates
DOP-PCR	Using of copy number assessment in single cell. Quick, No need normalization	Low coverage of genome (10 %). Unable for single nucleotide variant (SNV) detection. High false positive and negative rate, low success rate
PER-PCR	The operation is simple, the quality of template DNA is low the minimum starting quality is up to 5 pg	Low output and poor fidelity
LM-PCR	High coverage, the yield is high, the quality of template DNA is low	Low uniformity, the template DNA is easy to be lost by multi step operation.
T-PCR	High amplification efficiency and product specificity	Low gene coverage
LIANTI	The coverage and uniformity of this method are higher than other methods. Low allele depletion rate and false positive rate.	Needs further study
PTA	High coverage and uniformity. It has high accuracy as well.	Needs Further study
META-CS	High success rate. High amplification uniformity	Needs further study

MDA: multiple-displacement amplification, MALBAC: multiple annealing and looping based amplification cycles, DOP-PCR: degenerate oligonucleotide-primed polymerase chain reaction, PER-PCR: primer extension preamplification polymerase chain reaction, LM-PCR: ligation mediated polymerase chain reaction, T-PCR: tagged random primer polymerase chain reaction, LIANTI: linear amplification via transposon insertion, PTA: primary template-directed amplification, META-CS: multiplexed end-tagging amplification of complementary stands.

**Table 5:** Methods for single-cell CTC transcriptome sequencing.

Methods	A Key technology	Reverse- transcription enzyme used	Reverse-transcript size
Tang's method	poly A tailing	Reverse transcriptase	0.5–3.0 kb
Smart-seq	Template -Switching	M-MLV reverse transcriptase (RT)	Full length
Quartz sequencing method	Suppressed polymerase chain reaction (PCR)	Reverse transcriptase	0.4–4.0 kb
STAT Sequencing method	Template Switching	Reverse transcriptase	Full length but only detect 5′ end
Quantitative single-cell mRNA-Seq	Template-Switching, unique molecular identifiers	Reverse transcriptase	Full length, but only detected 5ʹ-end
Cel-seq	In vitro transcription, barcoding	Reverse transcriptase	Only 3ʹ-end
Smart-seq^2^	Template-switching, Locked nucleic acid	M-MLV reverse transcriptase (RT)	Full length

### 4.2 CTC sequencing analysis

Whole genome sequencing analysis of CTCs could unveil the origin of tumours, tumour heterogeneity, and drug resistance phenotypes, thereby identifying the pathway of tumour development [44]. There are several methods available for CTCs sequencing such as hydro seq and EPISPOT & EPIDROP assay are single-cell sequencing technologies for CTCs sequence analysis [58]. Hydro seq platform is a high-efficiency contamination-free cell capture single-cell RNA-seq platform that utilizes sizes based single cell capture to the eliminate biases [44]. In addition, Sanger sequencing, next-generation sequencing (NGS), and Array Comparative Genomic Hybridization (aCGH) can be used for CTC analysis.

Sanger sequencing or NGS methods not only detect specific gene mutations in CTCs but also provide information regarding inter and intra-tumour heterogeneity. Also, these methods unveil differential genetic makeup between CTCs of primary and metastatic cancer [44]. For example, CTCs from primary breast cancer and metastatic breast cancer tissues can be differentiated by the Sanger sequence [44]. Despite Sanger sequencing remaining the gold standard, it has two key limitations; the detection of 10–20 % mutated alleles in wild-type backgrounds and the ability to investigate only a few genes at a time [48]. For example, the analysis of PIK3CA and EGFR in single viable CTCs from breast and lung cancer patients was analyzed using Sanger sequencing [58].

aCGH technology analyses the copy number variation (CNV) in primary tumour, metastases, and CTCs [44]. On the other hand, NGS can be applied for single CTC sequencing to get information about panels of genes, the whole exome, or genome-wide CNVs [48]. Nevertheless, an amplification procedure of the genome of a single cell is necessary to allow sequencing analysis and only either of the two alleles are going to be amplified at the same rate. This generates a much more complex situation than the original sample in NGS. Therefore, WGA with NGS may result in low or non-uniform coverage, allelic dropout events, and false-positive and false negative results due to insufficient coverage. These technical errors must be considered in NGS data analysis. To minimize these problems, it has been assessed that achieving high physical coverage of the targeted sequences is crucial for calling mutations at the same regions across multiple single cells [48]. Thus, CTC analysis can be performed in various ways and may need to be customized to have better results. Table 6 provides some examples of CTC analysis in several cancers.

**Table 6:** CTC Sequencing of several cancer types.

Cancer types	CTC enrichment methods	CTC Sorting method	WGA method	CTC Analysis
Breast	Cell search	DEPArray	Ampli1™ WGA kit	qPCR/aCHH/Sanger
Lung	Cell search	Micropipetting under microscope	MALBAC	NGS
Colorectal	Cell search	Micromanipulation	GenomePlex, single amplificationkit, cell whole Genome	sanger/NGS/aCHH
Melanoma	Dynabeads	Laser Capture Microdissection	No WGA	Sanger
Prostate	Erythrocyte Lysis	Micromanipulation	Genomeplex, Single Amplification kit,Cell whole Genome	NGS
Pancreas	Dynabeads	Nano velcro	REPLI-g single cell kit	Sanger
Colon	Oncoquick, cell search	DEPArray	Ampli1™ WGA kit,MALBAC	Sanger/Pyrosequencing/NGS
Melanoma	Dynabeads	Laser capture microdissection	No NGS	Nested PCR/Sanger

## 5 Clinical significance of CTCs sequencing

### 5.1 Diagnosis, characterization, and stratification of metastatic cancers

Liquid biopsies to obtain CTC are used for screening, diagnosis, and prognosis, as well as, predicting early relapse, treatment response, and resistance of many solid cancers [52,59]. Future clinical research should aim to determine the precise frequency and time points for CTC testing throughout the follow-up of patients with cancer for better management in clinical settings. The importance of liquid biopsy in the management of some cancers such as small-cell lung cancer (SCLC), for instance, is highly appreciated since obtaining tumour biopsies is difficult, especially during recurrence and after chemotherapy; therefore, having more immediate access to CTCs can provide important information in selecting therapeutic regimen in patients with SCLC.

CTC sequencing could be an effective and distinctive liquid biopsy tool to track the development of cancer and find somatic mutations that might have therapeutic or clinical significance by comparing insights of CTCs’ genetic profiling associated with the progression or remissions of the clinical traits before and after therapeutic intervention [60]. For example, single-cell *KRAS* sequencing of CTCs in patients with prostate cancer using NanoVelcro/LCM platform noted that CTCs count was associated with the presence of *KRAS* mutations [61]. They reported that mutant *KRAS* was detected in CTCs isolated from prostatic cancer (92 %) with more than ten CTCs count whereas none of the other hematopoietic cells analyzed from the same patient had shown any such mutations [61]. It is assumed that heterozygous loss of *KRAS* in patients having prostatic cancer with fewer than ten CTCs had a clear reduction rate of mutations. In addition, the detection of EpCAM, CK19, CK20, and MUC2 and the presence of mutations in *KRAS* in CTCs from peripheral blood samples can detect colorectal cancer [22].

Additionally, efforts to explore the genetics of cancer on a wide scale and clinical sequencing from specific cancers may benefit from CTC sequencing. The integrated procedure may also allow for long-term tracking of the genetic status of widely spread cancer, providing crucial information on tumour development, the spread of metastatic disease, and drug resistance [62]. Moreover, as CTCs commonly harbored mutations in several tumour-related genes, such as those involved in treatment resistance and phenotypic changes, thus, the ability of CTCs to evade targeted therapy may be unveiled by sequencing them [63]. For example, CTCs sequencing from various cancers identified reproducible and concurrent variations in different malignancies, which provide crucial insights regarding the origin of cancer along with the spread of the tumours [22,63]. This information could be used for cancer categorization [63]. Furthermore, sequencing of exosomes derived from primary, metastatic tissues and CTCs revealed that there are common concurrent mutations in patients with prostate cancer, indicating that the CTCs contained information on both the primary tumour and the genetic mutations that occur during metastasis [21]. Consequently, it is possible to infer the mechanism of tumour metastasis from the mutations discovered in CTCs. Also, by single-cell sequencing, dynamic monitoring of primary tumour cells, CTCs, and tumour metastatic cells can help to understand the key oncogenes and tumour suppressor genes of tumour patients as well as the variation in the genome accommodate during the evolving of tumour. This information is crucial for early tumour diagnosis, dynamic treatment monitoring, the discovery of drug-resistance mutations, and other personalized treatment decisions [64]. Moreover, single-cell sequencing provides more detailed information for tumour stratification than tumour classification based on a single biopsy, which could frequently be used to support early tumour identification.

Single CTC sequencing might improve the cytopathology specimens' diagnostic efficacy with poor cellularity [65]. Also, CTC sequencing suggests the existence of cancer stem cells, which are a subgroup of highly malignant tumour cells and resistant to standard therapies. However, to support these hypotheses, single-cell RNA sequencing on a large number of CTCs per case is necessary. Additionally, a comparison between the characteristics of CTCs from a larger cohort of patients, including those with or without metastatic tumour, to those of CTCs from a patient's own CTCs is necessary. Furthermore, investigation of the genetic characteristics of CTCs and metastatic sites overlap and functional experiments employing ex vivo cell cultures of CTCs or xenografts to understand the involvement of CTCs in the mechanism of metastasis are imperative.

### 5.2 Prognosis

Cancer is a genetic disease that results from various genetic and epigenetic changes in the genome, including copy number variations (CNVs), single nucleotide variations (SNVs), insertions/deletions (INDELs), chromosomal aberration, etc. [66]. In clinical practice, it might be difficult for most patients to do biopsies at various tumour sites. Genomic studies of a few or single CTC derived from peripheral blood can reveal the genetic/epigenetic (e.g. CNVs/SNV/INDEL) profiles of the patients and the information can be used for prognosis and cancer management in clinical settings. CTC numbers and their phenotypic/genotypic properties associated with clinicopathological factors of patients, thereby have the potential to be used as prognostic biomarkers in clinical applications. For example, genomic alterations in *ESR1, GATA3, CDH1*, and CCND1 of CTCs are interrelated and could act as predictors of poor prognosis in patients with breast cancer [67]. It was noted that a connection between the detection of CTCs and the release of circulating tumour DNA (ctDNA) into the blood is shown in patients with breast cancer. Also, mutations in *ESR1* in individual CTCs matched mutations found in ctDNA [67]. In addition, the prognostic significance of a single CTC sequencing has been reported in breast, colon, prostate, lung, and pancreatic cancers [60]. For instance, CTC sequencing by NGS in metastatic breast cancer identified 51 mutations in 25 genes with the highest number of somatic deleterious mutations in the *TP53* gene [68]. These mutations were associated with adverse prognoses of patients with breast cancers [68]. Another study identified the mutational hot spot of *PIK3CA* by CTCs sequencing in metastatic breast cancer patients using SNaPshot methodology. They detected the common mutations in exon 9/E545K and exon 20/H1047R of the *PIK3CA* gene that has clinical relevance such as drug resistance against receptor tyrosine-protein kinase targeted therapy [62].

Mutations of *PIK3CA*, *RB1*, and *TP53* genes were detected in single CTCs derived from patients with lung cancer. Patients harboring these mutations exhibited drug resistance against erlotinib [69]. Other studies suggest that androgen receptor (AR) alterations in CTCs, particularly antigen receptor amplification and expression of the splice variant AR-V7, are associated with poor response to androgen deprivation therapy [[70], [71], [72]]. Furthermore, commonly altered genes include *AR* (androgen receptor), *ERG* (ETS-related gene), *c-MET* (tyrosine-protein kinase MET), *PTEN* (phosphatase and tension homolog deleted on chromosome 10), and PI3K/AKT signaling pathway genes in CTCs correlated with the development of castrate-resistant prostate cancer [[70], [71], [72]]. Whole-exome sequencing of CTCs in metastatic prostate cancer found mutational variants in several genes that shared between CTCs and matched metastatic biopsies, which are associated with metastatic castration-resistant prostate cancer [73].

Additionally, mutational variants of *EGFR, KRAS, and PIK3CA* genes are identified by CTCs sequencing in patients with colorectal cancer, which are associated with therapeutic response to *EGFR*-targeted therapy in patients [60]. Identification of mutations in *KRAS* in single CTCs associated with stage III patients with colorectal cancer and correlated with shorter disease-free survival [22].

*KRAS, TP53,* and *SMAD4* are the most frequently mutated genes in pancreatic cancer, and targeting them may be a promising treatment for cancer [64,74].

Ultimately, CTC sequencing could be a significant and novel method for monitoring the development of cancer and identifying somatic mutations with potential therapeutic implications that developed or eliminated before and after treatment. Thus, the molecular characterization of CTCs is essential to improving the prognostic specificity of CTC assays and investigating therapeutic targets. The first crucial step in CTC sequencing is obtaining enough cells for library preparation and sequencing. Nevertheless, based on different separation techniques and cancer types, different inferences might be made from diverse research. The clinical stage is also linked to the number of CTCs gathered from patients, and some cancer types tend to produce more CTCs than other cancer types. Although there are some debatable results, it is frequently the case that individuals with advanced cancer or metastatic lesions have higher CTC counts. Thus, when using single-cell CTC sequencing information as a prognostic indicator, these factors need to be considered.

Additionally, detection of CTC itself could be potential prognostic indicator in patients with cancers. For example, a meta-analysis incorporating 6825 cancer patients revealed the prognostic value of CTCs in patients with early stages and metastatic breast cancer [75]. In addition, number of CTCs act as an independent predictor of overall survival (OS) for castration-resistant prostate cancer [76]. It was noted that CTC in individuals were grouped as either Favorable (<5 CTC/7.5 mL) or Unfavorable (≥5 CTC/7.5 mL) based on their CTC number. OS was enhanced for the favorable group. Shorter OS was seen in the unfavorable group both before and after therapy. Furthermore, better OS has been linked to the change from the unfavorable group prior to therapy to the favorable group following it. Conversely, lower survival has been linked to the shift from the favorable to the unfavorable group after therapy. In addition, studies have demonstrated that the CTC number is a more accurate predictor of prostate cancer prognosis than PSA decline [[76], [77], [78]]. In order to categorize patients with similar CTC trajectory patterns across the course of treatment, Magbanua et al. created a novel latent mixture model. They also discovered that serial CTC analysis can be used to further categorize patients with poor prognoses into different prognostic categories [79]. Throughout the lengthy evolution of cancer, the dynamic alterations of CTCs may serve as a surrogate prognostic biomarker. As single-cell sequencing technologies continue to improve and become more accessible, it is reasonable to anticipate that in the future, CTC genomic/transcriptional profiles will function as a superior prognostic marker, providing biological data that is more detailed and directly associated with prognosis.

### 5.3 Predictive

Beyond enumeration alone, molecular studies of CTCs may offer predictive implications along with prognostic significance. A predictive biomarker provides information regarding the impact of therapeutics. By identifying therapeutic targets, resistance mechanisms, and the likelihood of response to therapeutics, predictive biomarkers based on the molecular properties of CTCs help physicians for optimization of therapeutic management for individual patients [80]. By sequencing, genomic abnormalities (e.g., genetic mutations, chromosomal translocations/rearrangements, and copy number variations) could be detected in CTCs, which can operate as predictive biomarkers to direct the use of targeted therapeutics with action only against cancers containing specific alterations. Because these molecular errors in tumours may show drug-targeting opportunities and resistance mechanisms, it is important to highlight that these genetic changes exhibit substantial intra- and interpatient heterogeneity, as well as discordance between original tumours, metastatic deposits, and CTCs [80,81].

With an increasing focus on targeted therapy, it is important to interrogate CTCs for targetable alterations, and a treatment decision based on the molecular signature of the CTCs might be more beneficial to the patients. Unlike conventional cytotoxic therapies, it is increasingly important to identify suitable pharmacodynamics and predictive biomarkers for targeted therapy [82]. A treatment choice based on the molecular signature of the CTCs may be more advantageous for the patients given the growing emphasis on targeted therapy and the need to examine CTCs for targetable mutations. It is becoming more crucial to find appropriate pharmacodynamics and predictive indicators for targeted therapy as opposed to standard cytotoxic medications [82]. For example, a study reported CTC’s roles as an early predictive biomarker for therapeutic response and the efficacy of HER2-targeted therapy in HER2-positive cancer with HER2-expressing CTCs. They noted that lapatinib, as a HER2-targeted therapy in combination with chemotherapy against chemotherapy alone in the patient group, is more effective and provide better clinical outcome [82]. Overall, the findings imply that tumour cell heterogeneity can be detected in CTCs, potentially offering a source of prognostic data beyond simple enumeration. Clinical applications relating to risk assessment and treatment response prediction may be made possible by this molecular knowledge. Another study described CTCs in localized prostate cancer and analyzed AR protein expression along with whole genome of a single CTC. They reported that CTCs with high AR protein expression are linked to the course of metastatic disease and AR-V7 expression in CTCs predicts a higher response to second-generation antiandrogen therapy [19]. Additionally, a higher heterogeneity for AR gene mutations and splicing variants was identified in CTCs by sequencing, which is associated with the therapy resistance of patients with prostate cancers [60]. This finding suggests that heterogeneity in the signaling pathway may be contributed to the treatment failure.

The advantage of CTC-based copy number aberration (CNA) analysis is that it is unaffected by typical cell contamination and has the potential to predict the therapeutic response more precisely. For example, Zhe et al. identified a subset of CNA from single CTC sequencing and created a CNA score that could forecast therapeutic efficacy for patients with SCLCs. They reported a relationship between CTC-based CNA profiles and the effectiveness of first-line chemotherapy (etoposide plus platinum) patients with SCLC. They identified the correlation in SCLC (n = 41) patients who completed the first-line chemotherapy using leave-one-out cross-validation and noted that a group of 10 CNA regions was strongly correlated with remission duration time (time from chemotherapy discontinuation to relapse) [83].

RNA-seq analysis of CTCs derived from breast cancer patients could act as a liquid biopsy that could predict the possible precursors of metastatic spread of the disease [84]. This study reported the complete transcriptome profiling of CTCs in patients with non-metastatic breast cancer and demonstrated that RNA-seq of CTCs could provide better insights regarding which CTC may allow for metastasis spread, thus, CTCs might be treated with molecularly targeted therapeutics [84]. Given the highly heterogeneous tumour biology of CTCs, it appears unlikely that single marker-based assays or limited panel multi-marker assays could characterize the tumour biology of CTCs to identify opportunities for targeted therapies. Instead, entire pathways should be investigated until suitable targets are known a priori. To anticipate treatment targets, this information might be used in conjunction with DNA sequencing [20].

Several WGS investigations on a single CTC may appear impracticable now. However, as sequencing technologies advance and costs go down, this strategy may prove helpful in figuring out how heterogeneity in an underlying malignancy evolves dynamically. Genomic instability in cancer has been identified as a possible roadblock to customized oncology care given its connection to treatment resistance. Recently, it has been proposed that this instability may cause a bigger creation of neoantigens, enhancing the efficacy of immunotherapy. This novel method of dynamic heterogeneity characterization may prove useful in therapeutic settings in the future [85]. As there is a significant degree of heterogeneity between individual CTCs from the same patient's HER2 expression and *PIK3CA* mutation status, combining CTC enumeration with mutational characterization at the single-cell level should aid patients with metastatic breast cancer in making better therapy decisions [86].

## 6 Conclusion

In this review, we presented different strategies or techniques for CTC sequencing as well as their clinical implications. Accumulating information indicated that CTC sequencing can be effectively employed as a liquid biopsy tool to examine the range of somatic variations and changes in gene expression in primary, metastatic, and recurrent cancers. The somatic variations could be employed to track the development or to comprehend the development of intra-tumour heterogeneity. The choice of target therapy could be the most significant clinical implication of CTC sequencing, which is for customized medicine or so-called precision medicine in patients with cancers. CTC capture and single-cell sequencing have both advanced. The creation of a more thorough cancer genesis and evolution model in the future could provide more translation clinical outcomes for better management of patients. In addition, through CTC sequencing, further novel biomarkers, or possible pharmacological targets for preventing or treating drug resistance, stopping cancer spread, or treating cancer with better efficacy could be seen in the near future.

## Funding

No specific funding was achieved for this project.

## Authors’ contributions

Data collection, analysis, and draft of the manuscript AA, MM, THP; preparation of figure and table AA, MM, KMR; editing the manuscript MN, AKL; supervision and editing the final draft FI.

## Ethics approval

Not applicable.

## Inform consent

Not applicable.

## Data availability

Data included in article/supplementary material/referenced in article.

## Declaration of competing interest

The authors declare that they have no known competing financial interests or personal relationships that could have appeared to influence the work reported in this paper.
